# Belief of having had unconfirmed Covid-19 infection reduces willingness to participate in app-based contact tracing

**DOI:** 10.1038/s41746-020-00357-5

**Published:** 2020-11-06

**Authors:** Patrik Bachtiger, Alexander Adamson, Jennifer K. Quint, Nicholas S. Peters

**Affiliations:** 1 National Heart and Lung Institute, Imperial College London, London, UK; 2 Imperial College Healthcare NHS Trust, London, UK

**Keywords:** Epidemiology, Infectious diseases

## Abstract

Contact tracing and lockdown are health policies being used worldwide to combat the coronavirus (COVID-19). The UK National Health Service (NHS) Track and Trace Service has plans for a nationwide app that notifies the need for self-isolation to those in contact with a person testing positive for COVID-19. To be successful, such an app will require high uptake, the determinants and willingness for which are unclear but essential to understand for effective public health benefit. The objective of this study was to measure the determinants of willingness to participate in an NHS app-based contact-tracing programme using a questionnaire within the Care Information Exchange (CIE)—the largest patient-facing electronic health record in the NHS. Among 47,708 registered NHS users of the CIE, 27% completed a questionnaire asking about willingness to participate in app-based contact tracing, understanding of government advice, mental and physical wellbeing and their healthcare utilisation—related or not to COVID-19. Descriptive statistics are reported alongside univariate and multivariable logistic regression models, with positive or negative responses to a question on app-based contact tracing as the dependent variable. 26.1% of all CIE participants were included in the analysis (*N* = 12,434, 43.0% male, mean age 55.2). 60.3% of respondents were willing to participate in app-based contact tracing. Out of those who responded ‘no’, 67.2% stated that this was due to privacy concerns. In univariate analysis, worsening mood, fear and anxiety in relation to changes in government rules around lockdown were associated with lower willingness to participate. Multivariable analysis showed that difficulty understanding government rules was associated with a decreased inclination to download the app, with those scoring 1–2 and 3–4 in their understanding of the new government rules being 45% and 27% less inclined to download the contact-tracing app, respectively; when compared to those who rated their understanding as 5–6/10 (OR for 1–2/10 = 0.57 [CI 0.48–0.67]; OR for 3–4/10 = 0.744 [CI 0.64–0.87]), whereas scores of 7–8 and 9–10 showed a 43% and 31% respective increase. Those reporting an unconfirmed belief of having previously had and recovered from COVID-19 were 27% less likely to be willing to download the app; belief of previous recovery from COVID-19 infection OR 0.727 [0.585–0.908]). In this large UK-wide questionnaire of wellbeing in lockdown, a willingness for app-based contact tracing over an appropriate age range is 60%—close to the estimated 56% population uptake, and substantially less than the smartphone-user uptake considered necessary for an app-based contact tracing to be an effective intervention to help suppress an epidemic. Difficulty comprehending government advice and uncertainty of diagnosis, based on a public health policy of not testing to confirm self-reported COVID-19 infection during lockdown, therefore reduce willingness to adopt a government contact-tracing app to a level below the threshold for effectiveness as a tool to suppress an epidemic.

## Introduction

Coronavirus disease 2019 (COVID-19), caused by severe acute respiratory syndrome-coronavirus 2 (SARS-CoV-2), has acutely incapacitated health systems, but there is growing concern that COVID-19 will be a long-lasting and fluctuating pandemic^1–3^. In the absence of an effective vaccine, the so-called non-pharmacological interventions have become the vanguard for reducing viral transmission by suppressing contact rates in the population^4,5^. These include physical distancing, decontamination and hygiene measures—as well as case identification and isolation with contact tracing and quarantine.

The UK government launched its COVID-19 contact-tracing programme, the National Health Service (NHS) Test and Trace Service, at the end of May 2020^6^. An initial pilot programme integrating an app developed by NHSX, the unit responsible for setting national technology policy in the NHS, proved unsuccessful for technical reasons. The UK government continues to develop its strategy for contact tracing, intent on launching an alternative smartphone app, which will run in the background using low-energy Bluetooth technology, before winter. Users will be notified to self-isolate if they have been in close proximity for more than 15 min with someone known to be COVID-19 positive, while maintaining anonymity.

A phone app is cheap, scalable and can help guide resource utilisation for the most effective disease control, but is critically dependent on levels of uptake and use by the population^7,8^. Germany^9^, South Korea^10^, Singapore^11^ and Hong Kong^12^ have all deployed app-based contact-tracing systems and to date have experienced comparatively lower fatality rates^13^. Notably, these were not only introduced much earlier in their national pandemics than the UK, but most importantly, as part of their national public health policies, were all introduced in the context of wide availability of diagnostic COVID-19 swab testing to confirm or refute the diagnosis in suspected community cases. This is in stark contrast with UK’s ‘stay-at-home’ policy of not testing to confirm or refute self-reported COVID-19 infection during the lockdown period, and there is inadequate understanding of the public’s willingness to participate in an app for contact tracing for COVID-19, and how this public health policy of staying at home untested may influence this willingness.

The exact proportion of engagement required for effectiveness is not yet known; the only modelling thus far addressing this question comes from a report submitted to NHSX by the Oxford University Big Data institute, suggesting effective epidemic suppression with 80% of all smartphone users using the app, or 56% of the population overall^14^. However, potential determinants of this willingness to participate, such as age, sex, healthcare service utilisation, impact of changing government rules on lockdown on wellbeing and perceptions of own experience of COVID-19, remain unknown.

## Results

### Participant selection

After excluding those aged <18, 13,095/47,679 (27.5%) individual responses were recorded, of which 12,452 (26.1%) were within the predetermined time frame (consort diagram in Supplementary Fig. 1). A further 18 participants did not answer the contact-tracing question and were excluded, leaving a total of 12,434 (26.1%) participants included in the analysis. Compared to excluded and non-responders, timely respondents included a larger proportion of males (43.0 vs 36.6%, *p* < 0.001) and were older on average (mean age 55.2 (SD 15.0) vs mean age 45.0 (SD 15.2), *p* < 0.001). A map of where CIE registrants live according to the first three letters of their postcode is included in Supplementary Fig. 2, highlighting that this is a UK-wide population. Summary Table 1 reports baseline characteristics and associations between responses to the question on contact tracing and other measured variables.

**Table 1:** General characteristics and questionnaire responses of the study population.

Variable	Total (*N* = 12,434), *N* (%)	Yes (*N* = 7503, 60.3%), *N* (%)	No (*N* = 2132, 17.1%), *N* (%)	Not sure (*N* = 2799, 22.5%), *N* (%)	*p*
Age					
Mean (SD)	55.2 (15.0)	55.1 (15.0)	55.6 (15.7)	55.0 (14.7)	0.321
Gender					
Male	5346 (43.0)	3263 (43.5)	970 (45.5)	1113 (39.8)	<0.001
Female	7088 (57.0)	4240 (56.5)	1162 (54.5)	1686 (60.2)	
Ease of understanding of new government rules; 1 = very difficult, 10 = very easy					
1–2	1494 (12.0)	753 (10.0)	390 (18.3)	351 (12.5)	<0.001
3–4	2238 (18.0)	1221 (16.3)	472 (22.1)	545 (19.5)	
5–6	2856 (23.0)	1667 (22.2)	471 (22.1)	718 (25.7)	
7–8	3347 (26.9)	2192 (29.2)	434 (20.4)	721 (25.8)	
9–10	2454 (19.7)	1650 (22.0)	355 (16.7)	449 (16.0)	
Missing	45 (0.4)	20 (0.3)	10 (0.5)	15 (0.5)	
Effect of new government rules on mood					
Better	1737 (14.0)	1205 (16.1)	226 (10.6)	306 (10.9)	<0.001
No change	6722 (54.1)	4098 (54.6)	1092 (51.2)	1532 (54.7)	
Worse	3917 (31.5)	2163 (28.8)	802 (37.6)	952 (34.0)	
Missing	58 (0.5)	37 (0.5)	12 (0.6)	9 (0.3)	
Effect of new government rules on anxiety					
Better	793 (6.4)	546 (7.3)	90 (4.2)	157 (5.6)	<0.001
No change	7018 (56.4)	4336 (57.8)	1136 (53.3)	1546 (55.2)	
Worse	4590 (36.9)	2601 (34.7)	896 (42.0)	1093 (39.0)	
Missing	33 (0.3)	20 (0.3)	10 (0.5)	3 (0.1)	
Effect of new government rules on fear					
Better	993 (8.0)	679 (9.0)	118 (5.5)	196 (7.0)	<0.001
No change	6584 (53.0)	4036 (53.8)	1110 (52.1)	1438 (51.4)	
Worse	4821 (38.8)	2771 (36.9)	894 (41.9)	1156 (41.3)	
Missing	36 (0.3)	17 (0.2)	10 (0.5)	9 (0.3)	
Displaying COVID-19 symptoms in past week which require 7 days isolation according to the NHS					
Yes	999 (8.0)	598 (8.0)	185 (8.7)	216 (7.7)	0.446
No	11,435 (92.0)	6905 (92.0)	1947 (91.3)	2583 (92.3)	
Patient has tested positive for COVID-19					
Yes	57 (0.5)	31 (0.4)	14 (0.7)	12 (0.4)	0.328
No	12,377 (99.5)	7472 (99.6)	2118 (99.3)	2787 (99.6)	
Patient is awaiting a test result for COVID-19					
Yes	76 (0.6)	52 (0.7)	10 (0.5)	14 (0.5)	0.349
No	12,358 (99.4)	7451 (99.3)	2122 (99.5)	2785 (99.5)	
Patient has tested negative for COVID-19					
Yes	463 (3.7)	294 (3.9)	76 (3.6)	93 (3.3)	0.333
No	11,971 (96.3)	7209 (96.1)	2056 (96.4)	2706 (96.7)	
Patient has taken a test for COVID-19^a^					
Yes	588 (4.7)	372 (5.0)	98 (4.6)	118 (4.2)	0.274
No	11,846 (95.3)	7131 (95.0)	2034 (95.4)	2681 (95.8)	
Patient has not taken a test for COVID-19, but thinks that they have had it and recovered					
Yes	600 (4.8)	325 (4.3)	124 (5.8)	151 (5.4)	0.005
No	11,834 (95.2)	7178 (95.7)	2008 (94.2)	2648 (94.6)	
Patient has received any healthcare contact since the start of lockdown					
Yes	6494 (52.2)	3898 (52.0)	1105 (51.8)	1491 (53.3)	0.454
No	5940 (47.8)	3605 (48.0)	1027 (48.2)	1308 (46.7)	

Table presents number of participants (*N*) and percentage of each category unless otherwise indicated. Variables are presented as a total and stratified according to patients’ responses to the contact-tracing app question. *P* value for categorical variables represents the chi-squared test for difference between groups, and for continuous variables represents a one-way analysis of variance test.

^a^Eight participants took more than one test for COVID-19; therefore, the total *N* for this question is less than the total *N* of positive/pending/negative combined.

### Overall willingness, privacy concerns and responses by sex and age

Overall, 60.3% of participants responded ‘yes’ to being willing to participate in app-based contact tracing, with 17.1% responding ‘no’ and 22.5% responding that they were unsure. Of those who responded ‘no’, 67.2% stated that this was due to privacy concerns, 21.9% stated that they did not have a smartphone or appropriate device and 10.9% stated that they did not feel able to download the app.

**Fig. 1:**
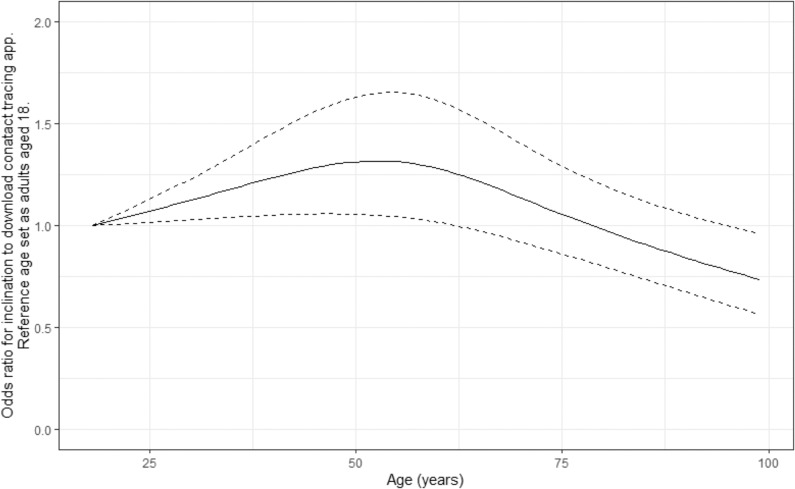
Odds ratio for the effect of age on the inclination to download a contact-tracing app, using a univariate logistic regression model with a restricted cubic spline transformation applied to age. Model uses 3 knots applied at the 0.1, 0.5 and 0.9 quantiles of the age distribution^30^. Solid line represents odds ratios against a reference of age 18. Dotted lines represent 95% confidence intervals. Model formula on log odds scale: 0.904 + 9.76e^−03^ * age − 8.392e^−06^ * (age − 33.0)^+3^ + 2.024e^−05^ * (age − 57.0)^+3^ − 1.185e^−05^ * (age − 74.0)^+3^.

Responses for yes and no did not differ significantly by sex (78.5% of females responded ‘yes’ compared to 77.1% in males after the removal of those who responded ‘not sure’), although females were more likely to respond ‘not sure’ than males. Responses were similar in all age groups from ages 18–79, ranging from 59.7 to 61.7%; however, only 53.0% of those aged 80 and above responded ‘yes’ to downloading the app, and this age group were more likely to report being unable to download an app or not having a suitable mobile device (Table 2). Figure 1 shows the univariate relationship between age and likelihood to download a contact-tracing app modelled using a restricted cubic spline analysis. Not being willing to participate on the grounds of privacy concerns was inversely associated with age.

**Table 2:** Breakdown of responses to app-based contact tracing by age group.

Age cat.	Response to app-based contact tracing					
	‘No—I do not feel able to do this’	‘No—I do not have a smartphone/appropriate device’	‘No—I have privacy concerns’	‘Not sure’	‘Yes’	Total
18–29	5 (0.8)	6 (1.0)	100 (16.4)	129 (21.1)	370 (60.7)	610
30–39	20 (1.1)	12 (0.7)	283 (15.6)	401 (22.1)	1097 (60.5)	1813
40–49	39 (2.1)	18 (1.0)	262 (13.8)	444 (23.5)	1129 (59.7)	1892
50–59	41 (1.5)	54 (2.0)	360 (13.0)	647 (23.4)	1658 (60.1)	2760
60–69	63 (2.1)	134 (4.5)	291 (9.8)	678 (22.7)	1816 (60.9)	2982
70–79	46 (2.3)	164 (8.2)	123 (6.2)	432 (21.6)	1231 (61.7)	1996
80+	18 (4.7)	79 (20.7)	14 (3.7)	68 (17.8)	202 (53.0)	381
*P* value	<0.001	<0.001	<0.001	0.398	0.876	

*P* value represents chi-squared test for trend for age.

### Univariate logistic regression

The results of the univariate logistic regression models are shown in Table 3. There was no association between age and a willingness to participate except in those aged above 80, who were 45% less likely to show a willingness to participate compared to those aged 18–29 (OR 0.55 [CI 0.40–0.75], 64.5 vs 76.9%) after exclusion of ‘not sure’ responses. Likewise sex, being tested for COVID-19, receiving a positive or negative test result or awaiting results, and reporting COVID-19 symptoms (cough, fever, anosmia) were not significantly associated with a willingness to participate. If new government rules led to a worsening mood, anxiety or fear participants were 15–28% less likely to respond ‘yes’ to willingness to download an app for contact tracing (OR 0.72 [CI 0.65–0.80], 73.0 vs 79.0%; OR 0.76 [CI 0.69–0.84], 74.4 vs 79.2%; OR 0.85 [CI 0.77–0.94], 75.6 vs 78.4%). A low understanding of government advice was associated with less willingness to download the app compared to those with moderate understanding (understanding reported 1–2/10 vs 5–6; OR 0.55 [0.47–0.64], 65.9 vs 78.0%).

**Table 3:** Associations between each variable of interest and willingness to download a contact-tracing app (Yes vs No).

Variable	Univariate analysis (odds ratios with 95% CI)	Multivariable analysis (odds ratios with 95% CI)*
Age (reference: 18–29)		
30–39	1.04 (0.81 to 1.33)	1.07 (0.83 to 1.38)
40–49	1.06 (0.83 to 1.35)	1.08 (0.84 to 1.38)
50–59	1.09 (0.86 to 1.38)	1.10 (0.86 to 1.40)
60–69	1.12 (0.88 to 1.41)	1.05 (0.82 to 1.33)
70–79	1.11 (0.87 to 1.41)	1.02 (0.79 to 1.30)
80+	0.55 (0.40 to 0.75)	0.50 (0.36 to 0.70)
Female	1.08 (0.98 to 1.19)	1.11 (1.00 to 1.24)
Ease of understanding of new government rules (reference: 5-6)		
1–2	0.55 (0.47 to 0.64)	0.56 (0.48 to 0.66)
3–4	0.73 (0.63 to 0.85)	0.74 (0.64 to 0.86)
7–8	1.43 (1.23 to 1.65)	1.37 (1.18 to 1.59)
9–10	1.31 (1.13 to 1.53)	1.24 (1.06 to 1.46)
Effect of new government rules on mood (reference: no change)		
Worse	0.72 (0.65 to 0.80)	0.90 (0.79 to 1.03)
Better	1.42 (1.22 to 1.67)	1.16 (0.97 to 1.38)
Effect of new government rules on anxiety (reference: no change)		
Worse	0.76 (0.69 to 0.84)	0.97 (0.84 to 1.11)
Better	1.59 (1.27 to 2.02)	1.24 (0.96 to 1.62)
Effect of new government rules on fear (reference: no change)		
Worse	0.85 (0.77 to 0.94)	1.07 (0.95 to 1.21)
Better	1.58 (1.29 to 1.95)	1.26 (1.02 to 1.58)
Displaying covid-19 symptoms in past week which require 7 days isolation according to the NHS	0.91 (0.77 to 1.09)	1.00 (0.83 to 1.19)
Patient has tested positive for COVID-19	0.63 (0.34 to 1.22)	–
Patient is pending a test result positive for COVID-19	1.48 (0.79 to 3.10)	–
Patient has tested negative for COVID-19	1.10 (0.86 to 1.44)	–
Patient has taken a test for COVID-19	1.08 (0.87 to 1.37)	1.08 (0.86 to 1.38)
Patient has not taken a test for COVID-19, but thinks that they have had it and recovered	0.73 (0.59 to 0.91)	0.73 (0.59 to 0.91)
Patient has received any healthcare contact since the start of lockdown	1.00 (0.91 to 1.11)	1.03 (0.93 to 1.14)

Table presents results for univariate logistic regression analyses and multivariable logistic regression adjusted for every other variable in the model.

Multivariable analysis performed on 9512 patients. Those who answered unsure (*N* = 2799) or were missing data for any other variable (*N* = 123) were not included in the analysis. Univariate analysis performed with the variable of interest as the only predictor in the model. Multivariable analysis adjusted for every other variable in the model. Only patients responding yes/no to receiving a test were included in the model due to low numbers in the groups testing positive and awaiting a test result.

### Multivariable logistic regression

The results of the multivariable logistic regression are shown in Table 3. Multivariable analysis showed that difficulty in understanding government rules around lockdown was strongly associated with being less willing to download the app (understanding 1–2/10 OR 0.57 [CI 0.48–0.67]; understanding 3–4/10 OR 0.744 [CI 0.64–0.87]). Those who indicated that they found it easier to understand government advice were more likely to indicate that they would download the app (7–8/10 vs 5–6 OR 1.37 [1.18–1.59], 9–10/10 vs 5–6 OR 1.24 [1.06–1.46]).

Belief of having previously had and recovered from COVID-19 was associated with being 28% less likely to be willing to participate (OR 0.72 [0.59–0.91]) in app-based contact tracing (Table 3) with 72.4% of those who believed that they had had and recovered from COVID-19 being willing to participate in contact tracing compared to 78.1% who did not. In the multivariable analysis, being female was associated with being willing to participate in contact tracing after adjusting for the effect of every other variable in the model (OR 1.11 [1.00–1.24]). There was moderate multicollinearity between changes in the effect of the new government rules on mood, anxiety and fear (VIF range 1.59–2.27).

## Discussion

This study reports questionnaire responses from 12,434 participants from across the UK, measuring determinants of willingness to participate in the anticipated NHS app for COVID-19 contact tracing. Overall, 60.3% of respondents were willing to participate in app-based contact tracing, with 22.5% unsure. Among participants answering ‘no’, 67.2% stated that this was due to privacy concerns. Worsening mood, fear and anxiety were associated with reduced willingness to participate in app-based contact tracing only by univariate analysis. Multivariable analysis showed that difficulty in understanding government rules was associated with a decreased inclination to download the app, with those scoring 1–2 and 3–4 in their understanding of the new government rules being 45 and 27% less inclined to download the contact-tracing app, respectively, whereas scores of 7–8 and 9–10 showed a 43 and 31% respective increase. Those reporting an unconfirmed belief of having previously had and recovered from COVID-19 were 27% less likely to be willing to download the app.

The principal finding of this study that overall 60% are willing to participate in app-based contact tracing is close to the estimated 56% of the total population, but far less than the estimated 80% of smartphone users, needed for the app to have beneficial impact on an epidemic^14^. Multivariate analysis showed a robust positive association between the likelihood of downloading the app and the participant’s understanding of government policies. This finding is potentially actionable: health policy orientated to improving the UK population’s understanding of government recommendations could boost uptake of app-based contact tracing. Mood, fear and anxiety are themselves determined by multiple complex factors, supported by these only being associated with reduced willingness to participate by univariate analysis but not after adjusting for confounders in the multivariate model.

Part of the challenge of evaluating potential uptake of digital public health interventions is inadequate sampling from those with health conditions—i.e. patients—who as an at-risk group^15,16^ stand to benefit most from participation in app-based contact tracing. An online poll in May 2020 by Opinium surveyed 2002 participants, with half (53%) stating they would be likely to download a contact-tracing app, while 21% would be unlikely to^17^. A further, non-representative poll of 730 participants suggested 73% were willing to download an app^18^.

This is the first instance of using the questionnaire functionality across the NHS’ largest patient-facing electronic health record (CIE) to collect, at scale, responses from a group of patients from across the UK. Other, conventional online questionnaires/poling platforms are characterised by selection bias, unknown denominators, inherently more digitally literate participants^19^ and limited external validity^20^. Importantly, the distribution of our CIE participants was not skewed towards younger age groups indicating that the concept referred to by others of the ‘digital divide’ by which older participants are thought incapable of participating in health initiatives with a digital element^21^, is not reflected in this study of users. This study therefore highlights the opportunities for the CIE as a tool for population health surveillance, both in the short-term of the COVID-19 pandemic and as the NHS continues with its digital transformation agenda^22^.

The timing of the questionnaire coincided with changes in government announcements of lockdown policy which for many created substantial uncertainty, and this might be expected to reduce motivation, and possibly trust, as an explanation for the observed association between unwillingness for app-based contact tracing and difficulty understanding government advice.

The UK adopted a public health policy during lockdown of instruction to stay at home if symptomatic of COVID-19 unless becoming very unwell with it^13,23,24^, resulting in a large number of people who believe they have had COVID-19 but without confirmatory testing. Participants in this questionnaire who reported the unproven belief of having had COVID-19 are less willing to participate on account of believing they may have immunity, and may be further disincentivized from using the app to avoid the risk of being instructed to self-isolate for 14 days.

There are several limitations to this study. Due to the time-sensitive need to deliver and inform health policy, an approach involving, for example, Delphi studies and psychometric evaluation of questions was not feasible. This would have enhanced the validity of the results. On this basis, we ensured that the questions posed underwent academic and clinical peer review and testing on patient participants before being administered. These results are only indicative; whether participants stick to their response when faced with wide release and accompanying messaging from the government to download the app is uncertain. However, previous studies have shown good correlation between declared survey responses and subsequent behaviour^25–27^. Twenty-two per cent of respondents were ‘not sure’ about their willingness, but this response was not further qualified to identify underlying reasons. Patients registered on the CIE without internet, who could not log in to their CIE account, or were incapable of understanding or responding will be underrepresented, though such potential biases will have been mitigated by the large sample size. This study will by default have included many ‘shielded’ patients, identified and advised by the NHS to stay at home at all times due to their disease profile placing them at higher risk of adverse outcomes from COVID-19^28^, for whom attitudes to participation in app-based contact tracing will have its own considerations but are no less important.

In conclusion, poor understanding of government rules around lockdown and belief of having had COVID-19 decrease willingness to participate in app-based contact tracing. Using the largest patient-facing EHR in the NHS as an effective and timely questionnaire tool, we have revealed the role of uncertainties in both government messaging and not testing suspected COVID-19 infection in reducing willingness for app-based contact tracing and the importance of eliminating uncertainty in lockdown and virus-testing policies.

## Methods

### Study participants

Participants in this study were individuals with a previous healthcare event or encounter, for example, hospital admission, outpatient appointment, medical investigation at a London-based tertiary NHS Trust (Imperial College Healthcare NHS Foundation Trust). Any such encounter triggered the creation of a digital record on the NHS Trust’s Care Information Exchange (CIE)^29^, the UK’s largest patient-facing electronic health record (EHR), displaying, for example, appointments, clinical letters, blood results, accessible to patients after registration with an email address. On the date on which the data were extracted (27th May 2020) the CIE held the records of 47,679 patients aged 18 years or older.

### Questionnaire design

The CIE has several functionalities for enhancing direct patient care, including the ability to create bespoke questionnaires. Invitation to respond to questionnaires is notified by email, with a direct web link for completion within a patient’s CIE record. The data analysed in this study were derived from a single questionnaire that was part of a longitudinal, weekly series implemented at the beginning of lockdown as a direct care tool for patients to keep track of their wellbeing.

### Timing of questionnaire

The questionnaire was sent out on Friday 15th May 2020, five days after the UK government changed its messaging around lockdown from ‘Stay Home, Protect the NHS, Save Lives’ to ‘Stay Alert, Control the Virus, Save Lives’, accompanied by the easing of some lockdown restrictions^28^. Responses submitted after Monday 18th May 2020 were excluded to minimise recall bias and ensure questions referring to ‘in the last week’ were not misinterpreted and were pertinent to the events of that week.

### Questionnaire content

The questionnaire used for this study included the addition of a question to measure a participant’s willingness to participate in app-based contact tracing, alongside established questions that formed part of the longitudinal weekly questionnaires for patients to track their wellbeing. Questionnaire items were developed by a clinical and academic collaboration with expertise in questionnaire design and qualitative analysis and were reviewed by a panel of patients before being finalised. Data relevant to analysis were: age, sex, understanding of changing government rules on lockdown; effect of changing government rules on lockdown on mood, anxiety and fear; experience of COVID-19 symptoms in last week, belief of previous COVID-19 illness and recovery, testing status for COVID-19, and healthcare contact since the start of lockdown. The full questionnaire is included in Supplementary Note 1.

### Data and consent

The Data Protection Office (DPO) of Imperial College Healthcare NHS Trust approved this study with ethical approval not required. The CIE, including the questionnaire, is a tool for direct care at the Trust, therefore as per DPO guidance consent for de-identified data analysis was not required. Participants were informed that their responses would be analysed to help inform local and national health policy and were free to opt out.

### Data analysis

All data were analysed using R (version 3.6.2). Questions with a response in the form of a five-point scale (a lot worse, a little worse, no change, a little better, a lot better) had their responses simplified to a three point scale (worse, no change, better) to aid interpretation of the results and account for low numbers in some categories. Age was categorised into 10 year age bands between 18–29 and 80+ to aid interpretation of a non-linear relationship between age and response to contact tracing. Participants who marked any one of the available options for healthcare encounters were classed as having any healthcare contact. Participants who marked themselves as having a new or worsened cough, a fever that was measured or unmeasured with a thermometer, or anosmia, were classed as displaying COVID-19 symptoms. Participants who stated that they had received any test for COVID-19 (positive/pending/negative) were classed as having received a test for COVID-19. Participants who stated that they had not received a test but thought that they had recovered from COVID-19, and additionally stated that they had received a test, were classed as having had a test and removed from the ‘no test but think recovered’ group (*N* = 8). Descriptive statistics are reported for the dataset as a whole and broken down according to response to inclination to download an NHS contact-tracing app (yes/no/unsure). Differences between groups were assessed using chi-squared tests for categorical variables and analysis of variance tests for continuous variables. *P* values <0.05 were considered statistically significant. Relationships between inclination to download the app (yes vs. no) and each variable of interest were then assessed using univariate and multivariable logistic regression, with unadjusted odds ratios and odds ratios adjusted for every other variable in the model provided alongside 95% confidence intervals. A non-linear relationship between age and inclination to download the app was additionally visualised using restricted cubic spline analysis. Due to low numbers reporting testing positive or awaiting their test result, only the binary variable of receiving a test result yes/no was included in the multivariable analysis. Multicollinearity was assessed by calculation of the variance inflation factor (VIF), with variables with a VIF > 5 (indicating substantial multicollinearity) removed from the model.

### Reporting summary

Further information on research design is available in the Nature Research Reporting Summary linked to this article.

## Supplementary information

Supplementary Information

Reporting Summary

## Data Availability

Imperial College Healthcare NHS Trust is the data controller. The datasets analysed in this study are not publicly available but can be shared for scientific collaboration by contacting the corresponding author and subject to meeting requirements of the institution’s data protection policy.

## Code availability

All analyses were conducted in R (version 3.6.2). Code used in the analysis is available upon request from the corresponding author.
