# Supplementary material for: Belief of having had unconfirmed Covid-19 infection reduces willingness to participate in app-based contact tracing
Source: NPJ Digit Med. 2020 Nov 6;3:146. doi: 10.1038/s41746-020-00357-5 (PMC7648058; doi:10.1038/s41746-020-00357-5)

Supplementary Materials

Supplementary Note 1. Care Information Exchange questionnaire.

Care Information Exchange

UPDATE: NEW QUESTIONS ADDED SINCE CHANGE IN GOVERNMENT ADVICE

LoC-19 - COVID-19 Wellbeing in Lockdown – Imperial College Healthcare NHS Trust

We would like to once more thank the large number of patients who continue to provide valuable responses to this questionnaire. Further updates have been made based on patient feedback and evolving government advice around lockdown.

We have updated the questions for this week and removed several to make things easier.

SECTION 1 -- Key questions and updates (please answer all)

SECTION 2 -- Important questions around symptoms and healthcare usage (please answer all)

This serial questionnaire will enable you to keep track of your physical and mental health during this difficult period, and will allow Imperial College Healthcare NHS Trust to understand how best to respond to this pandemic for our patients.

You will be able to see your responses in your CIE record and these will inform vital research by Imperial College & NHS Trust. Your information will be treated with full confidentiality within the Care Information Exchange, and no personally identifiable data will be provided to research staff. We are aware of other surveys being conducted by Imperial that may involve some of you – and so are doubly grateful for your hard work which really will make a difference to our understanding and ability to help all round.

Please ignore subsequent email invitations if you do not wish to participate. Please note that we will not respond to any submissions on an individual level. If you are unwell please seek medical attention as normal. Please send any enquiries or comments about the questionnaire to Professor Nicholas Peters at nicholas.peters2@nhs.net

----

SECTION 1 - Key questions and updates (please answer all)

How easy or difficult has it been to understand the latest government rules and advice on lockdown?

1 = very difficult

2

3

4

5

6

7

8

9

10 = very easy

Has the latest change in government rules significantly changed your lockdown routine?

No

Yes

How do you feel physically today?

1 = worst

2

3

4

5

6

7

8

9

10 = best

How is your mood today?

1 = worst

2

3

4

5

6

7

8

9

10 = best

MOOD

Compared with before lockdown, how is your mood?

A lot worse

A little worse

No change

A little better

A lot better

How is the latest change in government rules and advice affecting your mood?

A lot worse

A little worse

No change

A little better

A lot better

SLEEP

Compared with before lockdown, how is your sleep?

A lot worse

A little worse

No change

A little better

A lot better

How is the latest change in government rules and advice affecting your sleep?

A lot worse

A little worse

No change

A little better

A lot better

GENERAL ANXIETY

Compared with before lockdown, how are your feelings of general anxiety?

A lot worse

A little worse

No change

A little better

A lot better

How is the latest change in government rules and advice affecting your feelings of general anxiety?

A lot worse

A little worse

No change

A little better

A lot better

FEAR OF CORONAVIRUS

Compared with the beginning of lockdown, how are your levels of fear of coronavirus?

- A lot worse
- A little worse
- No change
- A little better
- A lot better

How is the latest change in government rules and advice affecting your fear of coronavirus?

- A lot worse
- A little worse
- No change
- A little better
- A lot better

HOUSEHOLD RELATIONSHIPS

Compared with before lockdown, how are your household relationships?

- A lot worse
- A little worse
- No change
- A little better

A lot better

How is the latest change in government rules and advice affecting your household relationships?

A lot worse

A little worse

No change

A little better

A lot better

EXERCISE

Compared with before lockdown, how are your levels of exercise?

A lot worse

A little worse

No change

A little better

A lot better

How is the latest change in government rules and advice affecting your levels of exercise?

A lot worse

A little worse

No change

A little better

A lot better

PHYSICAL FITNESS

Compared with before lockdown, how is your feeling of physical fitness?

A lot worse

A little worse

No change

A little better

A lot better

How is the latest change in government rules and advice affecting your feeling of physical fitness?

A lot worse

A little worse

No change

A little better

A lot better

WEIGHT

Compared with before lockdown, how is your weight?

A lot worse

A little worse

No change

A little better

A lot better

How is the latest change in government rules and advice affecting your weight?

A lot worse

A little worse

No change

A little better

A lot better

CONTACT-TRACING

Contact-tracing is the process of identification of persons who may have come into contact with an infected person. If the government advised you to download an NHS app for your phone (like the one being tested on the Isle of Wight) to help with contact-tracing for coronavirus, would you do this?

Yes

Not sure

No - I do not have a smartphone / appropriate device

No - I have privacy concerns

No - I do not feel able to do this

SECTION 2 - Important questions around symptoms and healthcare usage (please answer all)

In the last week, have you had a cough?

- No
- Yes -- I have a new, dry cough
- Yes -- I have a new cough bringing up phlegm
- Yes -- but I usually have a cough like this
- Yes -- my usual cough has worsened

In the last week, have you experienced unusual shortness of breath compared to what's normal for you?

- No
- Yes -- Mild symptoms, slight shortness of breath during ordinary activity
- Yes – Significant symptoms, breathing is comfortable only at rest
- Yes -- Severe symptoms, breathing difficult even at rest

In the last week, have you had a fever and did you take your temperature?

- I have NOT felt feverish
- I have felt feverish but did not check my temperature
- I felt feverish and my temperature was BELOW 38 degrees Celcius
- I felt feverish and my temperature measured ABOVE 38 degrees Celcius

In the last week, have you experienced any of these other symptoms? Please tick all that apply

- [ ] I haven't had any new symptoms
- [ ] Loss of sense of smell
- [ ] Loss of appetite (skipping meals)
- [ ] Diarrhoea
- [ ] Vomiting
- [ ] Fatigue
- [ ] Sneezing
- [ ] Chest pain / tightness
- [ ] Sore throat
- [ ] Runny nose
- [ ] Itchy eyes
- [ ] Headache
- [ ] Joint pain / aches

In the last week, has anyone in your household had a new cough or fever?

Not applicable

No

Yes

Have you or anyone in your house been tested for coronavirus? Please tick all that apply

- [ ] No testing
- [ ] I have not been tested -- BUT I think I have already had coronavirus and recovered
- [ ] I was tested - positive result
- [ ] I was tested - awaiting result
- [ ] I was tested - negative result
- [ ] Household member tested - positive result
- [ ] Household member tested - awaiting result
- [ ] Household member tested - negative result

Have you had any healthcare contact since the lockdown started? Please tick all that apply

- [ ] No
- [ ] Yes - remote appointment with my GP (phone/video)
- [ ] Yes - I attended my GP practice for an appointment
- [ ] Yes - I have had new contact with mental health services (remote or in person, including counselling)
- [ ] Yes - remote appointment with hospital (phone/video)
- [ ] Yes - I attended hospital for an appointment
- [ ] Yes - attended Accident and Emergency
- [ ] Yes -- I was admitted to hospital (not because of coronavirus)
- [ ] Yes -- I was admitted to hospital with symptoms of coronavirus

Thank you for your ongoing contribution to this important work.

Choose privacy

Choose privacy setting to determine who has access. For example, changing HIV to "General Health" means that everyone who has access to your general health record can see the diagnosis while changing it to "Sexual Health" means only those who have access to your sexual health record can see it.

(•) General health

Most of your health record is general health and includes information that most health professionals will use to deliver care

( ) Social care

Information about the care you receive from your local authority social care team, including disability funding. This is very useful in helping to manage home care services

( ) Mental health

Information about mental health conditions, for example diagnoses of anxiety, depression or schizophrenia. It is very useful for your GP to see such information alongside your mental health team

( ) Sexual health

Information about sexual health. This includes reproductive health and sexually transmitted infections

Cancel Submit

Imperial Health Charity

Supplementary Figure 1. CONSORT flow diagram. Detailing respondent selection included in the analysis.

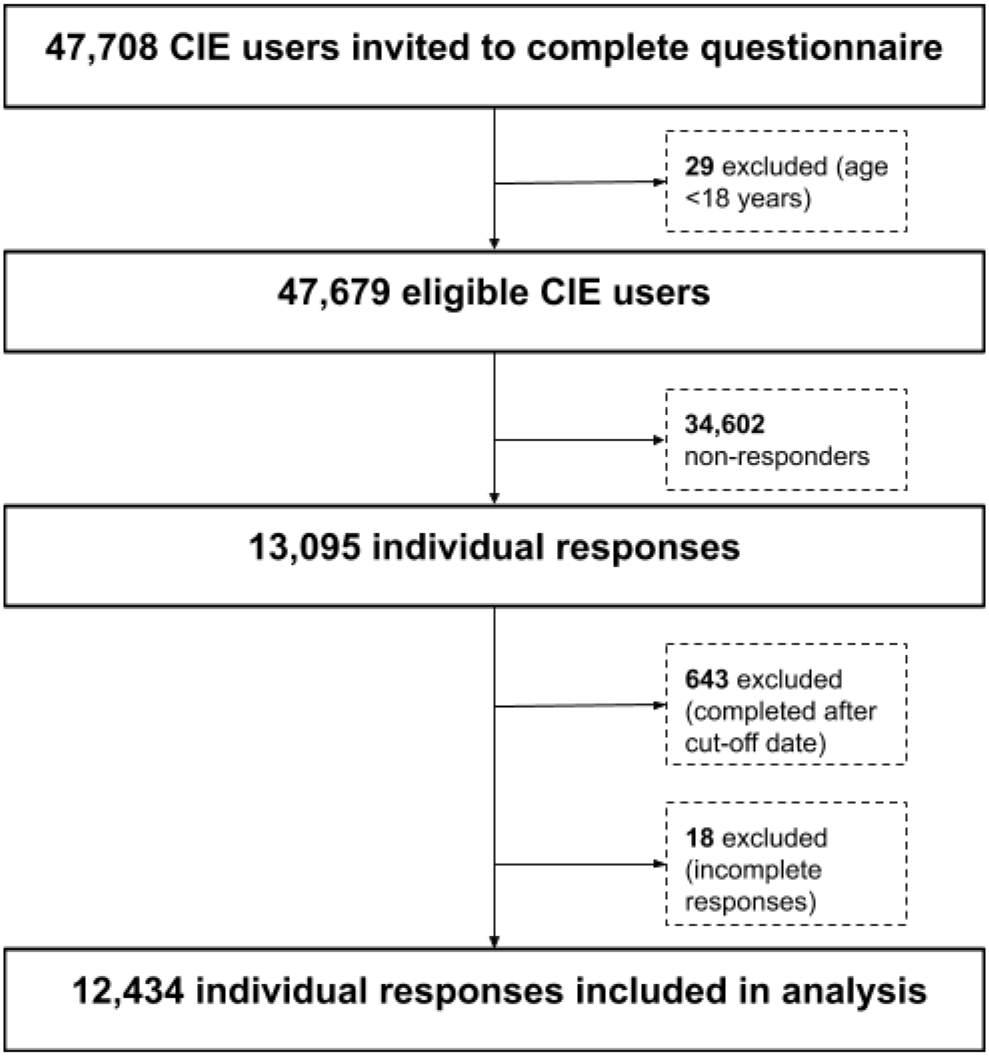

Supplementary figure 2. Map of registered CIE users by postcode. Each blue dot indicates at least one registrant living in that postcode. Extracted from Tableau May 5th 2020.

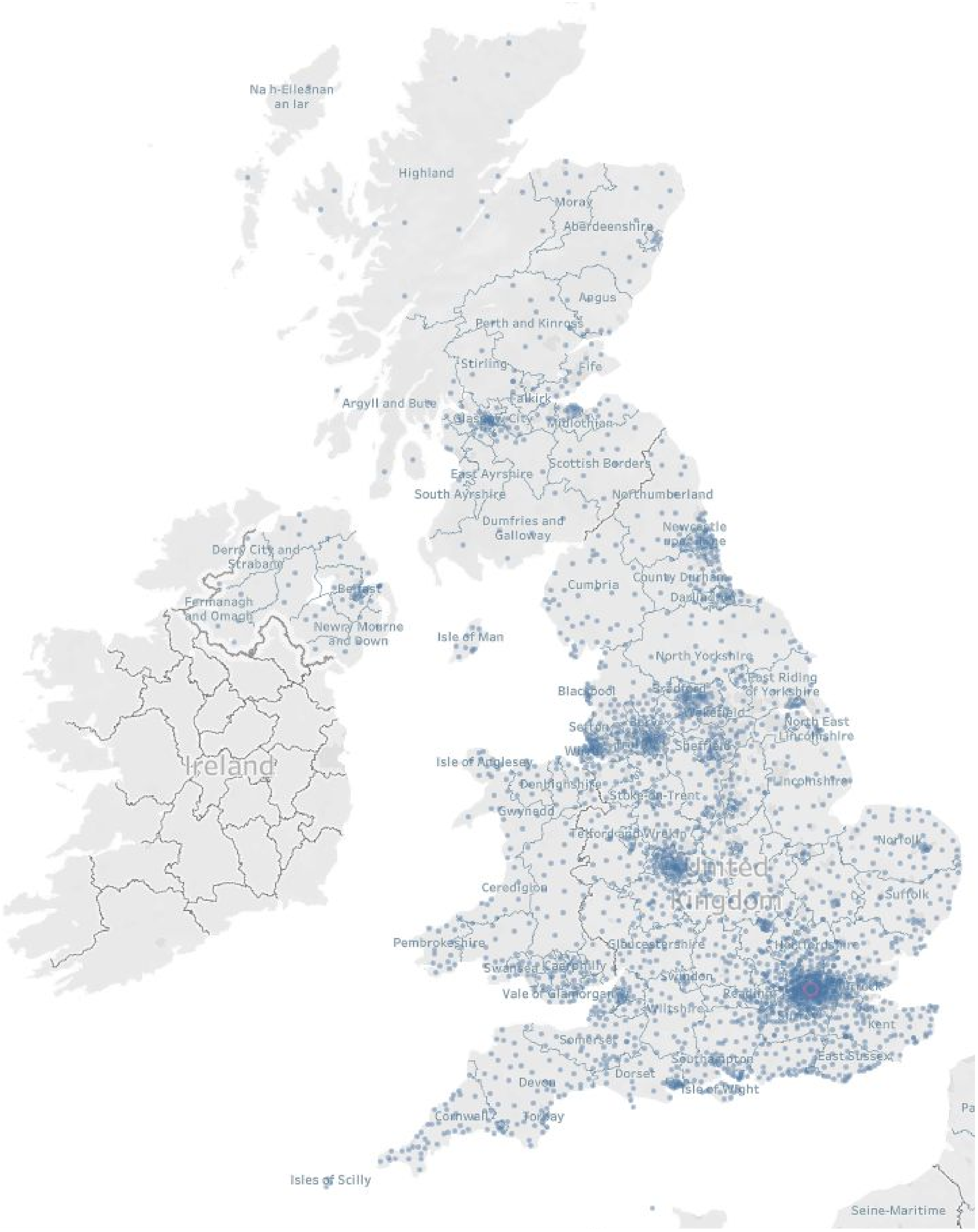

Supplement: Supplementary file 1 — Supplementary Information [file 41746_2020_357_MOESM1_ESM.pdf]
