# Supplementary material for: Belief of having had unconfirmed Covid-19 infection reduces willingness to participate in app-based contact tracing
Source: NPJ Digit Med. 2020 Nov 6;3:146. doi: 10.1038/s41746-020-00357-5 (PMC7648058; doi:10.1038/s41746-020-00357-5)
Supplement: Supplementary file 2 — Reporting Summary [file 41746_2020_357_MOESM2_ESM.pdf]

nature research

Corresponding author(s): Nicholas S Peters

Last updated by author(s): 2020/10/05

Reporting Summary

Nature Research wishes to improve the reproducibility of the work that we publish. This form provides structure for consistency and transparency in reporting. For further information on Nature Research policies, see our Editorial Policies and the Editorial Policy Checklist.

Statistics

For all statistical analyses, confirm that the following items are present in the figure legend, table legend, main text, or Methods section.

n/a	Confirmed	
☐	☒	The exact sample size (n) for each experimental group/condition, given as a discrete number and unit of measurement
☐	☒	A statement on whether measurements were taken from distinct samples or whether the same sample was measured repeatedly
☐	☒	The statistical test(s) used AND whether they are one- or two-sided<br>*Only common tests should be described solely by name; describe more complex techniques in the Methods section.*
☐	☒	A description of all covariates tested
☐	☒	A description of any assumptions or corrections, such as tests of normality and adjustment for multiple comparisons
☐	☒	A full description of the statistical parameters including central tendency (e.g. means) or other basic estimates (e.g. regression coefficient) AND variation (e.g. standard deviation) or associated estimates of uncertainty (e.g. confidence intervals)
☐	☒	For null hypothesis testing, the test statistic (e.g. F, t, r) with confidence intervals, effect sizes, degrees of freedom and P value noted<br>*Give P values as exact values whenever suitable.*
☒	☐	For Bayesian analysis, information on the choice of priors and Markov chain Monte Carlo settings
☒	☐	For hierarchical and complex designs, identification of the appropriate level for tests and full reporting of outcomes
☒	☐	Estimates of effect sizes (e.g. Cohen's d, Pearson's r), indicating how they were calculated

Our web collection on statistics for biologists contains articles on many of the points above.

Software and code

Policy information about availability of computer code

Data collection	All data were collected and stored in secure servers in encrypted csv files within Imperial College Healthcare NHS Trust
Data analysis	All data were analysed in R v 3.6.2 using established code. Code script used in this study is available upon request from the corresponding author.

For manuscripts utilizing custom algorithms or software that are central to the research but not yet described in published literature, software must be made available to editors and reviewers. We strongly encourage code deposition in a community repository (e.g. GitHub). See the Nature Research guidelines for submitting code & software for further information.

Data

Policy information about availability of data

All manuscripts must include a data availability statement. This statement should provide the following information, where applicable:

- Accession codes, unique identifiers, or web links for publicly available datasets
- A list of figures that have associated raw data
- A description of any restrictions on data availability

Imperial College Healthcare NHS Trust is the data controller. The datasets analysed in this study are not publicly available but can be shared for scientific collaboration by contacting the corresponding author and subject to meeting requirements of the institution’s data protection policy.

Field-specific reporting

Please select the one below that is the best fit for your research. If you are not sure, read the appropriate sections before making your selection.

☐ Life sciences ☒ Behavioural & social sciences ☐ Ecological, evolutionary & environmental sciences

For a reference copy of the document with all sections, see nature.com/documents/nr-reporting-summary-flat.pdf

Behavioural & social sciences study design

All studies must disclose on these points even when the disclosure is negative.

Study description	Observational study
Research sample	Registrants of the Imperial College Healthcare NHS Trust Care Information Exchange (CIE).
Sampling strategy	Participants responding to a questionnaire within their CIE record.
Data collection	Responses retrieved by direct download of encrypted csv files from CIE.
Timing	Questionnaire sent out May 15th 2020; data retrieved May 27th.
Data exclusions	Exclusion of responses later than 4 days after questionnaire was sent
Non-participation	After excluding those aged below 18 years, 13,095/47,679 (27.5%) individual responses were recorded, of which 12,452 (26.1%) were within the predetermined time frame. A further 18 participants did not answer the contact tracing question and were excluded, leaving a total of 12,434 (26.1%) participants included in the analysis.
Randomization	N/A

Reporting for specific materials, systems and methods

We require information from authors about some types of materials, experimental systems and methods used in many studies. Here, indicate whether each material, system or method listed is relevant to your study. If you are not sure if a list item applies to your research, read the appropriate section before selecting a response.

Materials & experimental systems

n/a	Involved in the study	
☒	☐	Antibodies
☒	☐	Eukaryotic cell lines
☒	☐	Palaeontology and archaeology
☒	☐	Animals and other organisms
☒	☐	Human research participants
☒	☐	Clinical data
☒	☐	Dual use research of concern

Methods

n/a	Involved in the study	
☒	☐	ChIP-seq
☒	☐	Flow cytometry
☒	☐	MRI-based neuroimaging
